# Adaptation to Lactose in Lactase Non Persistent People: Effects on Intolerance and the Relationship between Dairy Food Consumption and Evalution of Diseases

**DOI:** 10.3390/nu7085309

**Published:** 2015-08-13

**Authors:** Andrew Szilagyi

**Affiliations:** Division of Gastroenterology, Department of Medicine, Jewish General Hospital, McGill University School of Medicine; 3755, Chemin de la Cote-Ste-Catherine Rd, Rm E110, Montreal H3T 1E2, QC, Canada; E-Mail: aszilagy@jgh.mcgill.ca; Tel.: +1-514-340-8144; Fax: +1-514-340-8282

**Keywords:** lactase, lactose, digesters, maldigesters adaptation

## Abstract

Dairy foods contain complex nutrients which interact with the host. Yet, evolution of lactase persistence has divided the human species into those that can or cannot digest lactose in adulthood. Such a ubiquitous trait has differential effects on humanity. The literature is reviewed to explore how the divide affects lactose handling by lactase non persistent persons. There are two basic differences in digesters. Firstly, maldigesters consume less dairy foods, and secondly, excess lactose is digested by colonic microflora. Lactose intolerance in maldigesters may occur with random lactose ingestion. However, lactose intolerance without maldigestion tends to detract from gaining a clear understanding of the mechanisms of symptoms formation and leads to confusion with regards to dairy food consumption. The main consequence of intolerance is withholding dairy foods. However, regular dairy food consumption by lactase non persistent people could lead to colonic adaptation by the microbiome. This process may mimic a prebiotic effect and allows lactase non persistent people to consume more dairy foods enhancing a favorable microbiome. This process then could lead to alterations in outcome of diseases in response to dairy foods in lactose maldigesters. The evidence that lactose is a selective human prebiotic is reviewed and current links between dairy foods and some diseases are discussed within this context. Colonic adaptation has not been adequately studied, especially with modern microbiological techniques.

## 1. Introduction

The ability or inability of adults to digest the disaccharide lactose into its components, glucose and galactose, divides humanity into two Mendelian inherited phenotypes. The dominant emergence of lactase persistence (LP) some 7500–10,000 years ago occurred almost simultaneously in Europe, Africa and the Middle East [1,2]. Those populations that domesticated milk animals and consumed their milk eventually showed higher frequencies of mutations associated with a higher expression of the enzyme lactase in adults [3]. Dominant mutations occurred in the lactase promoter region upstream from lactase phlorizin hydrolase on chromosome 2q21 [1,2] retaining intestinal lactase into adulthood. In ancient times, it is hypothesized that consumption of dairy foods may have added a nutritional advantage to such populations [4]. In arid conditions, drinking fluid milk may have allowed desert dwellers an additional source of water [3]. In northern Europe, populations exposed to low sunlight with low skin synthesis of vitamin D may have derived ability to consume more calcium through dairy products and avert risks of Rickets [5]. It is estimated that lactase underwent a strong and recent signal of positive selection [6].

In this review we will discuss the concept of “adaptation” to dairy foods (DFs). Although different DFs contain variable amounts of lactose [7] the emphasis is on the total content of lactose consumed, rather than individual components (milk, yogurt cheese and others). The reason is that it is lactose which may the adapting nutrient in lactase non persistent persons (LNP).

Adaptation refers to two processes. The first is the adaptation to milk drinking based on ancestral herding practices. The second, which is primarily discussed in this review, refers to lactose maldigesters (lactase non persistent people with less than 10 units/g of intestinal lactase; LNP) who despite their genetic status, regularly consume DFs and as a result have improved lactose handling. Colonic adaptation in LNP people likely results in altered microbiome and metabolome (discussed below). These alterations then may impact on health issues. Improved symptoms of lactose intolerance (LI) and the altered microbome are closely linked in this paradigm. Prebiotics and probiotics are briefly discussed to illustrate their potential in affecting various diseases. We then argue that lactose qualifies as a prebiotic. We finally discuss the variable effects of dairy foods (DFs) on different diseases and argue that the possible prebiotic lactose effect in LNP populations are not adequately explored. Colonic adaptation in LNP persons may introduce a confounder if LP/LNP is ignored, because the mechanisms of DF effects may be different in some diseases in the two populations.

There are differences in the way LNP persons handle lactose containing DFs. Firstly, countries with a high proportion of LNP populations consume less DFs than countries with high proportions of LP populations [8]. The second occurs after regular DF consumption and is associated with a reduction both in symptoms [9] and in bacterial hydrogen production specifically measured by the lactose breath hydrogen test [10]. These two latter features are linked. The less the symptoms of LI in LNP persons the greater the quantity of lactose containing DFs may be consumed. In turn, continued lactose intake may support the altered microbiome/metabolome in the long term.

Lactose containing products are perceived as primary culprits of gastrointestinal symptoms. Intolerance of lactose and hence many dairy foods have been at the forefront of this notion. A strict definition of LI endorsed by a National Institute of Health (NIH) conference on the topic requires the diagnosis to be based on a comparison with an inert placebo [11]. Table 1 outlines a number of definitions in the literature relating to lactose. These features need to be further clarified and may occur with or without LM.

**Table 1:** Terms related to lactose and its digestion.

Term	Interpretation
Lactase Persistent; LP	The dominant genetic trait in adults with
	continuous ability to digest lactose throughout adulthood
Lactase Non Peristent; LNP	The natural decline in intestinal lactase to <10 u/g
	of tissue which leaves adults with minimal ability to
	digest lactose
Lactase Deficiency; LD	Reduction of intestinal lactase enzyme from
	either genetic (LNP) or any secondary causes
	due to diseases of the proximal small bowel mucosa
Lactose Maldigestion; LM	Inability to digest lactose for any cause primary
	(LNP) or secondary causes resulting in undigested
	lactose reaching the colon
Lactose Intolerance; LI	Symptoms resulting from the ingestion of lactose
	including flatus, gas, bloating, cramps, diarrhea
	and rarely vomiting. Currently, symptoms must
	not be present when an inert placebo is exchanged
	for lactose
Lactose Sensitivity;	Symptoms with or without symptoms of LI and
	the systemic features depression, headache, fatigue

While the NIH definition may be relevant in clinical studies, it is unclear whether this definition matters to persons complaining of LI and consumption of DFs. The obsessive preoccupation with LI is attested to by almost 1.2 million Google search results on the internet dealing with this topic.

The symptoms of LNP persons are attributed to osmotically active undigested lactose which increases intestinal transit and then colonic metabolism of lactose. He *et al.* noted that while the rate of lactose digestion was similar in lactose tolerant and intolerant LNP subjects, the clearance of short chain fatty acids (acetate propionate and butyrate; SCFA) was reduced in the LI group [12].

Variables besides lactase deficiency can impact LI. Symptoms depend on dose of lactose, the solution lactose is dissolved into (water or milk with variable fat content) or whether other non DFs foods are consumed. DFs with lesser lactose content may be tolerated much better (e.g., yogurt, hard cheeses, DFs made with milk of lower lactose content and other fermented dairy products). Similarly, gastric and intestinal motility can influence symptoms [13].

In addition, diseases affecting the upper gastrointestinal tract (infections; Giardia, bacterial overgrowth, malabsorptive diseases like celiac disease of the brush border) can induce secondary maldigestion with onset of new LI. Whether small amounts of lactose in medicines induce symptoms has been debated [14,15]. The role of larger doses of lactose ingestion is discussed below.

The definition of LI has been somewhat clouded by the introduction of the controversial concept of “systemic” effects of lactose such as headache, fatigue and depression [16]. In addition lactose sensitivity (LS) has been coined in diseases like Crohn’s (an inflammatory bowel disease affecting any portion of the intestine), whose relationship to ethnicity appears to be non-existent [17]. LS however is also considered to be part of food intolerances in the FODMAP (Fermentable Oligo, Di Monosaccharide And Polyol) hypothesis [18]. In this hypothesis, several carbohydrates are responsible for classical symptoms of “intolerance”. In this way, LI may be misinterpreted and the mechanisms put forth by He *et al.* can be fulfilled even in LP subjects [12].

The relationship of LI to LM has been further eroded by the recognition in well-designed studies that people who believe themselves to be LI have difficulty in distinguishing verum from placebo [19,20,21,22]. Also, a nocebo effect was reported [23]. This term is applied to harmful/negative effects of an inert substance. A placebo effect could possibly explain improvement of symptoms with continued consumption of lactose [24]. Although adaptation through colonic metabolomic changes could be another factor in symptomatic improvement, this is unlikely to occur in LP persons.

## 2. Symptomatic Improvement with Regular Lactose Consumption in LNP People

Among LNP populations it was noted that despite initial symptoms with DFs, improvements occurred after some time [9]. At least two early reports were published from areas where LNP populations dominate, that symptoms from powdered milk dissipated after a month of continued consumption [25,26]. In a group of African Americans who were lactose intolerant, increasing lactose content over a 6–12 week period led to improved symptoms [27].

There are at least two physiological conditions where lactose intolerance may be modified in LNP persons. These are pregnancy and untreated thyroid disease. One study suggested that as pregnancy progresses, LI may improve [28]. Support for this finding was provided by another study which suggested that LI symptoms increased postpartum [29]. However, a third study reported that LI symptoms at any time in pregnancy were less severe than in a control group of non-pregnant African American women, but LM frequency did not change [30].

The mechanisms postulated for reduced symptoms were lactose adaptation by colonic bacteria as pregnancy progressed [28]. Indeed, pregnant women in a small sample did consume more DFs during pregnancy than after [29]. However, it was also postulated that prolonged intestinal motility expected with increasing serum progesterone levels could also contribute [29]. The hypothesis is that increased transit time reduces the quantity of lactose entering the colon, delaying its bacterial metabolism. A correlation between peak hydrogen, gut motility and LI symptom scores has been published [31]. However, an *in vitro* study in Caco2 cells (which mimic human intestinal cells) failed to show any stimulatory effect of gestational hormones on lactase [32]. The clinical findings from the first two studies are displayed in Table 2.

**Table 2:** Summary of altered lactose maldigestion and intolerance in pregnancy.

Author	No.	LM in	LM	LM	BH2 Auc	BH2* Auc	BH2* Auc
	Included	Preg N	Prepart	Postpart	Pregnancy	Prepart	Postpart
Villar [28]	114	62/118 (54%)	35/118 (29.7%)		116 ± 9.6 ppm ^+^	54 ± 7.3 ppm	

Szilagyi [29] ^+^	28	16/28 (57.1%)		20/28 (71%)	3816.9 ± 577.4 ppm		6490 ± 925 ppm

* = *p* < 0.01; ^+^ = Mean Total Symptom Score consisting of flatulence/gas, cramp/abdominal pain, bloat/distension and diarrhea; postpartum 7.7 ± 8.7 *vs.* prepartum 4.4 ± 1.3 (*p* = 0.07); LM = Lactose Maldigester, BH2 Breath Hydrogen Test, reported as total Auc area under the 3 or 4 h hydrogen curve; prepart or postpart = prepartum or postpartum; ppm = parts per million.

In the study of African Americans by Paige *et al.* [30], the authors reported no frequency change in LM at any time during pregnancy or postpartum compared with non-pregnant controls. However, examination of results of breath hydrogen in different groups shows numerical decrease in Auc (area under the curve) in LNP women during pregnancy compared with postpartum. The peak hydrogen during pregnancy occurs at the 5th hour (of an 8 hour) test while the peak hydrogen post-partum occurs at the 4th hour. This suggests that LM women produced less hydrogen during pregnancy than postpartum. Reanalysis of peak hydrogen postpartum compared to early pregnancy was higher and statistically significant (two sided *t*-test, *p* = 0.037). The combination of reduced symptoms and reduced hydrogen response to lactose challenge suggest improved lactose handling in pregnancy.

In untreated hyperthyroidism, symptoms of lactose intolerance in LNP persons may be aggravated [33]. In hypothyroidism, they may be less severe [34]. However, in treated hypothyroidism, there is some suggestion that lactose intolerance may interfere with proper therapy of the low thyroid state and lactose withdrawal may facilitate therapy [35]. The reasons for these discrepancies are not clear. In small animal studies, thyroxine increased intestinal lactase in embryogenesis [36] but later it inhibited intestinal lactase [37]. The above clinical observations could be explained by inhibition of human intestinal lactase but human studies are scarce [36]. The altered symptoms however may also be explained by motility changes noted in different thyroid states [38,39,40]. Rapid gastric emptying and increased intestinal transit could deliver larger amounts of lactose across the ileocecal valve, leading to increase in bacterial metabolism and symptoms [12].

Proof of principle that altered intestinal motility could alter LI in LNP persons was shown in a study using different doses of the antidiarrheal and anti-motility agent (Loperamide HCL, Imodium^®^ McNeil Consumer Healthcare, Fort Washington, PA, USA) [41]. Table 3 outlines the results.

**Table 3:** The effects of increasing doses of loperamide in men responding to a 50 g aqueous lactose challenge on three different days (after washout times) are shown. Reproduced from reference [41] with permission from John Wiley and Sons.

	Area under the curve for beath H_2_	Symptom Score (Max = 42)	Delayed Symtom Score (no max)	Lactose Intestinal Transit time (min)	Oral cecal Transit time (min)
Baseline (*n* = 16)	10,243 ± 1607	18.7 ± 3	8 ± 1.3	43.1 ± 6.8	56.9 ± 5.9
8 mg loperimide (*n* = 14)	8527 ± 1456.7	8.5 ± 2.1*	5.5 ± 1.5^†^	48.2 ± 7.4	82.1 ± 13.9*
12 mg Loperimide (*n* = 16)	7685 ± 935.6**	10.3 ± 2.4*	3.1 ± 1*	63.1 ± 9.7*	90.3 ± 11.1*

* *p* < 0.05 *vs*. baseline, paired t test; **p < 0.05 *vs.* baseline, Wilcoxon rank sum test; **^†^** One subject could not be contacted for a delayed symptom assessment.

Both the area under the hydrogen breath curve (50 g lactose challenge) and oral cecal transit time were significantly increased by the 12 mg dose compared with baseline. Similarly, the oral cecal transit time in response to the lactose load was prolonged significantly. Interestingly, the symptom score reduction did not show a dose response [41].

These two conditions highlight the complex interactions between symptoms, physiology of gastrointestinal motility. In both cases, the intestinal bacterial response to altered lactose delivery may also impact on clinical observations.

The altered intestinal bacterial response can also lead to improved symptoms. Bacterial metabolic response was first clinically shown in a study of 20 LNP subjects by Hertzler and Savaiano [10]. In a blinded crossover placebo (dextrose) studyk the typical breath hydrogen response to a 50 g lactose challenge was completely converted from that of a LM response to a LP response 16 days after consuming increasing daily lactose. In addition to normalization of hydrogenk these subjects also had less gas and bloating. Improvement to lactose challenge in LNP subjects has also been noted using the prebiotic lactulose as an adapter [42]. Furthermorek adaptation was applied using a derivative of lactose, galacto-oligosaccharide [43]. In this double blind randomized studyk galacto-oligosaccharide 15 g/day was given for 35 days to 85 LI/LNP subjects. Following this period of prebiotic consumption, DFs (unspecified) were reintroduced and participants were followed for an additional month. Both digestion and abdominal pain improved in at least half the subjects and were six times less likely to report lactose intolerance [43].

Regardless of the mechanisms, reduction of symptoms in LNP persons could increase DF consumption. There is very little evidence for this contention. Besides the study of Hertzler and Savaiano [10], a single case report of a 32 year old Sicilian male (expected LM status of 70%–80%) is published [44]. He had a completely normal breath hydrogen test with daily intake of 28.1 g of lactose from different DFs (approximate content in 500mL of milk). When he abandoned DFs for three weeks, a repeat 50 g lactose challenge revealed a classical LM breath pattern. Upon refeeding DFs, the hydrogen response curve diminished, although it was not as dramatic as at the beginning). Presence of “colonically adapted” subjects among LNP populations could modify effects of DFs compared with non DF consuming LNP or DF consuming LP persons. This hypothesis is further elaborated below.

## 3. Probiotics, Prebiotics and Health Benefits for the Host

This section deals with potential health benefits of probiotics and prebiotics. Probiotics are defined as live microorganisms that, when administered in adequate amounts, confer a health benefit to the host. Prebiotics are defined as predominantly maldigested carbohydrates which, upon reaching the colon, are selectively metabolized through fermentation by specific lactic acid producing bacteria (Bifidobacteria and Lactobacilli) altering the metabolism and exerting benefits to the host [45]. Either pre or probiotics could alter microbial flora and microbial metabolism and provides a prelude to a discussion on the possible prebiotic effects of lactose consumption in LNP persons.

In the last five years, there has been a growing interest in the relationship between the human microbiome and different diseases, as shown by the number of publications on the subject [46,47,48,49,50]. The microbiome contains over 10^14^ organisms outnumbering human cells by 10 to 1. Most reside in the lower intestine. The ability to evaluate microbial genetics and metabolism without identifying specific bacteria has provided a great advantage in understanding the complexities and metabolic interactions between microbes, other unicellular organisms and the host [46]. The role of bacterial distributions such as the ratio of the bacteroidetes to fermicutes in the development of such diseases as obesity, metabolic syndrome, Type 2 diabetes (T2D), the inflammatory bowel diseases, irritable bowel syndrome, atherosclerosis and others has emerged as being important to disease development [46,47]. The interaction of different bacteria leading to a state of eubiosis (normal bacterial distributions and interactions which have not been clearly established yet) can be disturbed leading to dysbiosis (abnormal bacterial distributions and interactions) and has been postulated in pathogenesis of different diseases [48,49].

The microbiome of the host develops from the time of birth and reaches an adult microflora by the second or third year of life [48]. Early interventions such as breast feeding or substitution of oligosaccharides impacts on early development [48]. The adult microbiome is generally stable and is dedicated to the individual host. However, a number of factors during life (e.g., antibiotics, diseases, breast feeding, post weaning and adult diet) can alter the basic flora. In fact, the dominance of Western (meat, animal fat, high sugar) diet compared to more carbohydrate and agrarian diets leads to predominant “enterotypes” with dominant Bacteroides or Prevotella organisms, respectively [49,50].

Feeding live probiotic bacteria (such as those in making yogurt) for better health was recognized for at least a century (first noted by E. Metchnikoff). Modern scientific interest in use of probiotics for health preceded exponentially active research on the microbiome. There are multiple beneficial functions provided by probiotics [47]. There are numerous studies on both gastrointestinal and non-gastrointestinal disorders in which probiotics have been examined for benefits. Among these, Rota virus related gastroenteritis [51], post antibiotic diarrhea prevention [52], recurrence of C. difficile infection [53], irritable bowel syndrome [54], adjunctive treatment for ulcerative colitis [55], pediatric atopy [56], possible maternal health of pregnant women [57,58], and type 2 diabetes [59] have been found to be benefited.

In the case of LI, the use of probiotics have been associated mostly with yogurt. The question is whether any long term benefits can be achieved by consumption of probiotics. In a small study of 27 patients by Almeida *et al.*, a yogurt containing *L. casei* Shirota and *B. breve* showed evidence of reduced breath hydrogen and symptom scores after four weeks of consumption compared with baseline. Furthermore, the beneficial effects seemed to last at least three months after probiotic cessation [60]. In another small study, the use of the eight probiotic combination, VSL#3 powder failed to improve breath hydrogen after a 50g lactose load at baseline and again at 17 days after regular probiotic consumption [61]. The reason for different outcomes is not clear but could relate to types of bacteria given, the length of consumption time and the vehicle in which the probiotic is supplied.

The concept of functional foods and prebiotics which lead to changes in the microbiome and its metabolic functions were introduced, principally by Gibson and Roberfoid [45]. Primary prebiotics are polymers of either fructose (fructo-oligosaccharides, oligofructose or long chain inulin) or galactose (Galacto-oligosaccharides). However, the disaccharide lactulose (fructose, galactose) has also been accepted as a prebiotic [62]. It is of note that neither probiotics nor prebiotics need to affect proportions of the microbiome on fecal analysis [63]. Nevertheless, microbial genes involved in carbohydrate metabolism are activated [64]. Prebiotics, unlike probiotics, have general effects on the microbiome and induction depends on existing species, strains and initial bacterial counts. There are geographic differences in dominant strains of bifidobacteria or lactobacilli responding to a prebiotic. For example, Young *et al.* found different dominant species of bifidobacteria in stools of neonates from Ghana, New Zealand and the United Kingdom. Species from Ghana exerted different effects on dendritic cells *in vitro* from those species found in neonatal stools from the other countries [65]. The effect of long term Western or agrarian diet on shaping different microflora has also been reported in children from European or African centers, respectively [66]. Prebiotics may be looked upon as undirected synbiotics because the specific strains of bacteria to be stimulated are not known in the individual. This contrasts with the combination of specific prebiotic and species of known strains combined as a “synbiotic” with expected health outcomes. Table 4 outlines mechanisms of prebiotic effects [67,68,69,70,71,72,73,74]. Table 5 summarizes clinical conditions where prebiotics have been used. In general, the effects are less robust than for probiotics but there are also fewer clinical trials [75,76,77,78,79,80,81,82,83,84,85,86,87,88,89,90].

**Table 4:** Several functions ascribed to prebiotics which may be beneficial to the host.

Effect	Reference
Increases Fecal bulk and Laxation	[67]
Reduction of Intestinal Transit time	[67]
Microbial Substrate leading to SCFA	[68]
Butyrate preferred colonic nutrient	[68]
Anti carcinogen and inflammatory SCFA	[69]
Immune Modulation	[70]
Selective Stimulation of Lactic acid bacteria	[71,72]
Lactic acids stimulate other butyrogenic producing bacteria, altering metabolome and metagenome	[72,73]
Facilitated Calcium and other electrolyte absorption	[74]

**Table 5:** Human Conditions in which Prebiotics have been evaluated. OS = oligosaccharides; DS = disaccharides; GOS = Galacto-oligosaccharides; FOS = Fructooligosaccharides; OFS = oligofructose; RCT = randomized controlled trial.

Gastrointestinal/Nongastrointestinal	Prebiotic	Outcome	Reference
Infectious Gastroenteritis	OFS	Amoebic Gastroenteritis	[75]
Antibiotic Associated Diarrhea	OFS/Inulin	No effect	[75]
Irritable Bowel Syndrome	GOS/FOS	Modest benefit	[76,77,78]
Inflammatory Bowel Disease	Mixed Prebiotics	Possible Maintenance in UC	[46,79]
Colorectal Cancer	lactulose	decreased poly pformation	[80,81,82]
Lactose Intolerance	DS/OS	Improved symptoms Bifidobacteria expand	[32,33,34]
Necrotizing Enterocolitis	FOS/GOS	noclinical benefit	[83]
Cirrhosis (hepatic encephalopathy)	lactose(LNP), lactulose	Improved coma grade	[85,86]
Constipation	Fiber	no specific prebiotics	[67]
Acute Upper Respiratory Infections	GOS/FOS CT	rate lower with diet	[75]
Obesity, Metabolic Syndrome	FOS/inulin	improved satiety	[87]
Diabetes Type 2	FOS/Inulin	improved glucose/insulin	[87]
Pediatric Eczema	GOS/FOS	reduced eczema	[88,89]
Pediatric Atopic Dermatitis	OS/Pectin RCT	reduced *vs.* control	[89]
Urinary Infections	lactulose	modest reduction	[90]

The mechanisms as to how prebiotic select for metabolic preference have been evaluated. Falony *et al.* found that different strains of bifidobacteria fall into four metabolic clusters depending on the rate these strains metabolize fructans of different degree of polymerization [71]. In comparing the ability of strains of Bifidobacteria with co cultures of Bacteroides the taiotaomicron, Falony *et al.* also found that different bifidobacteria strains had a metabolic advantage over other bacteria [72]. This experiment gives clues as to how prebiotics are selective for lactic acid bacteria.

Prebiotics serve as substrate for the production of short chain fatty acids (SCFA; acetate, propionate and butyrate). Of these, butyrate has been considered as the main metabolite for colonocytes and is potentially associated with multiple anti-cancer and immune modifying effects [68]. There are, however, few clear clinical benefits so far found for SCFA related to improved colonic inflammation [91,92].

The main metabolic products of bifidobacteria or lactobacilli are acetic and lactic acid. Duncan *et al.* reported that the presence of lactic acid in intestinal milieu stimulated the growth and metabolism of a number of butyrogenic bacteria (*E. haali* and *A. caccai*) which are not the typical butyrogens [73].This paper lends to the notion that prebiotics in fact orchestrate a metabolic change in the microbiome.

## 4. Microbial and Metabolomic Effects of Lactose

Regular lactose consumption reduces hydrogen output on hydrogen breath tests [10]. Reduced quantity of expired hydrogen is correlated with some symptomatic improvement [10,31] and is attributed to larger concentrations of “lactic acid” bacterial response. *In vitro* metabolism of lactose was found to be associated with expansion of both lactobacilli and bifidobacteria in fecal slurries [93,94]. Another *in vitro* study reported that lactose could reduce ammonia production. [95].

Ammonia is a bacterial species specific metabolic product and its production can be reduced by probiotics such as lacto or bifidobacteria.

After the introduction of the concept of prebiotics [45], it was reasoned that in LNP populations, regular consumption of lactose could be considered as a prebiotic [96]. Part of the rationale is the ready acceptance of the disaccharide lactulose (fructose and galactose) as a prebiotic. This sugar is universally malabsorbed and on reaching the colon stimulates the growth of bifidobacteria [70]. The possibility that lactose is a selective prebiotic rests on the finding that in humans lactase is not an inducible enzyme [97,98] and a single study which suggested that lactose intake in LP individuals results in some (0%–8% of ingested lactose) spilling into the colon [99]. However, to our knowledge, the observation was never repeated. Table 6 outlines studies which support the prebiotic nature of lactose.

**Table 6:** Evidence supporting a metabolic impact of lactose on the microbiome.

*In Vitro*	Reference
Hydrogen production is reduced and is not due to low pH	[100]
Bifidobacteria or Lactobacilli efficiently metabolize lactose	[93,94]
Lactose decreases bacterial ammonia production	[95]
In a model colon lactose increases growth of Bifidobacteria	[101]
A prebiotic Index for lactose has been calculated as 5.75	[102]
*In Vivo* (Animal)	
Lactose induces Lactobacilli in Colon (pigs)	[103]
Lactose enhances metabolome of microflora (rats) Human	[104]
Lactose improves hepatic encephalopathy in lactose maldigester cirrhotics	[84]
Lactose improves some features of lactose intolerance in lactose maldigesters	[10]
Measurable increase in fecal β- galactosidase after regular lactose or lactulose consumption	[10,42]
Lactose induced expansion of Lactobacilli and Bifidobacteria. Decreased Bacteroides and Clostridia species	[105]
Selective increase in fecal Bifidobacteria in lactose maldigesters but not lactose digesters after 2 weeks of lactose ingestion.	[106]

Several other *in vitro* studies showed that lactose induces the growth of bifidobacteria throughout a model colon [101]. Increase in bifidobacteria and lactobacilli is also accompanied by a proportional reduction of the genus Bacteroides and Clostridia. The ratio of the sum of the lactic acid bacteria to bacteroides and clostridia is called a prebiotic index [107]. This value is 5.75 for lactose and is comparable to lactulose and a number of other defined prebiotics [102].

*In vivo* clinical studies also support the prebiotic effect of lactose. For example, in a pig model, lactose induced species of lactobacilli while sucrose had no effect [103]. In a rat model, similarly, sucrose had no effect but lactose induced metabolic changes in the colonic microflora within 5 hours [104].

In clinical situations, the use of lactose to reverse hepatic encephalopathy in Mexican (LNP) cirrhotic patients has been reported [84]. In a study form Japan (a county populated by 90%–100% LNP), it was reported that there was significant growth of lactobacilli, a proportional increase in bifidobacteria and reduction in bacteroides and clostridia in subjects given lactose [105].This *in vivo* study mimics the *in vitro* derivation of the prebiotic index. In a small study, 23 LNP and 18 LP subjects were tested with a 25 g lactose load, hydrogen measured with a hydrogen monitor before and after consuming 25 g lactose twice a day for two weeks. Fecal counts of bifidobacteria increased modestly but significantly in LNP and not in LP participants. Although full adaptation was not achieved in this study, the differential effect of regular lactose consumption on LNP *versus* LP persons was shown as proof of principle [106].

In LNP persons, there is some debate as to how much lactose is tolerated. The NIH Consensus in 2010 considered that 12 g is tolerated by most LNP persons [11] and may be lower in some Asians [108]. However, this does not mean that lactose is not spilled at lower doses into the colon. When a hydrogen response is measured to different doses of lactose challenge, the test begins to become positive at 6 g [109]. As little as 5 g/day of short chain oligofructose may be enough to stimulate the microbiome [110]. On a regular basis, small amounts of lactose ingestion could alter the metabolomic and metagenomic features of the host and may impact on conditions listed in the section on probiotics and prebiotics. However, there is limited information on how quickly adaptation dissipates. In the study of Hertzler and Savaiano, the effects were dissipated in 72 hours [10].

Conclusions to be drawn from these last three sections are that: (1) consumption of DFs in LNP is different when lactose content exceeds a threshold; (2) such bacterial metabolism of lactose could reduce LI and alter the microbiome and its metabolism; (3) diet induced microbial/metabolomic changes may alter risks of different diseases evaluated for DF effects.

## 5. Effects of Dairy Foods on Diseases

This section outlines diseases studied in relation to DF consumption. Studies are complex because different aspects of DFs are analyzed in relation to complex diseases. Although several types of DFs are consumed, in general the impact of total DFs and in some cases milk (high lactose content, about 50 g/L cow’s milk) are emphasized in favor of individual products (e.g., cheese, cream, or fermented DFs lactose contents listed in reference [7]). Most of the studies discussed failed to evaluate any potential differences in lactose handling between LNP and LP.

Very few studies evaluate lactose content per se and almost none evaluate DF consumption along heterogeneous LP/LNP divides. In fact, a recent review considers lactose in milk as a cause for adverse events [111] but we argue that in LNP it may be a beneficial nutrient if adaptation by colonic bacteria occurs. An LNP person who regularly consumes lactose containing DFs and has few or no symptoms may be colonically adapted. The more adapted the individual, the more lactose consumption is possible and perhaps microbial alterations are sustained long term.

Impact of DFs on different diseases often demonstrate modest increased or reduced risks and depend on the hypothesized pathogenesis of the disease. In LNP populations, if the putative process involves the micobiome or a beneficial nutrient in DF, a prebiotic contribution through colonic adaptation may reduce risk (altered microbiome/metabolome or improved tolerance to increased consumption). In some diseases, the microbiome change may compensate for beneficial effects noted in DF consuming LP persons. In diseases where DF related pathogenesis may depend on other “toxic” factors, colonic adaptation could allow LNP persons to consume more DFs possibly accentuating risk. In general, however, LNP consumers of DFs fit into mid-range quantity intake [8].

Possible impacts of DFs may be inferred from ecological observations as well as clinical case-control or cohort studies. However, the direction of effect (harmful or beneficial) may differ between these observations. For example, ecological evaluation of the effect of annual per capita DF intake on a number of “Western” life style diseases was found to be different when patient level intake of DFs in the same diseases were examined [8].

DF intake is perceived to reduce risks of gastrointestinal cancers [112]. The best evidence is found for colorectal cancer. Several meta-analyses of DFs have shown protective effects against colorectal cancer [113,114]. The principal protective factor is hypothesized to be calcium which inhibits the proliferation of aberrant crypt foci in the colon. Studies of elemental calcium have provided evidence that high dose calcium is needed for protection against colorectal cancer and polyps [115]. Protection is afforded at levels which reach up to 1.2–1.3 g/day. Dairy foods contain the highest quantities of calcium [111]. In Western countries, the addition of Vitamin D to milk is also thought to protect against colorectal cancer [116,117], but vitamin D is generally less likely to be added in Africa, Asia and the Middle East.

**Figure 1:**
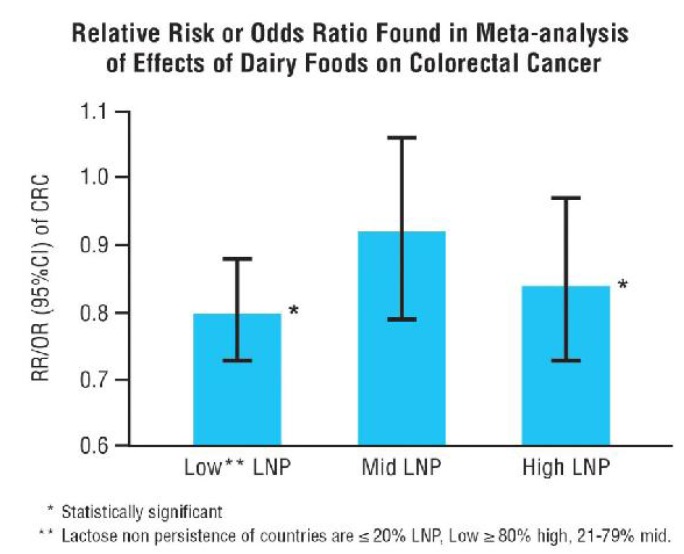
Bar graph represents the summary Odds Ratios or Relative Risks of the effect of dairy foods on colorectal cancer based on a meta-analysis of 67 studies [118]. In individual studies, the highest dairy food intakes was compared with the lowest intake. The three bars with 95% confidence intervals represent the outcome of regional meta-analyses of individual regional studies. The regions are divided according to percent lactase distribution (Lactase Persistent (LP)/Lactase Non persistent (LNP)) of the populations into low LNP (≤20%) (North America, Western Europe, Australia, N represents the number of studies 42) mid LNP (21%–79%; mean 50%) (South Europe, Latin America, N of studies 17) and high LNP (≥80%) (Asia, N of studies 8).Meta analyses include both case-control and cohort studies, although the original analyses also reported these types of studies separately. Modest but statistically significant reduction of colorectal cancer is noted in high (RR = 0.84, 95%CI = 0.73–0.97) and low LNP (RR = 0.80, 95% CI = 0.73–0.88) regions. In the mid LNP studies, a small non-statistically significant reduction in CRC is noted (RR = 0.92, 95% CI = 0.79–1.06). Modified figure reproduced from Szilagyi *et al.* [118] with permission from Francis & Taylor.

Examination of the effects of DF consumption on colorectal cancer reveal a curious geographic pattern. If case-control and cohort studies are grouped into regions with relatively high LP populations (Western LP dominant), high LNP populations (Eastern, Asian LNP dominant or mixed LP/LNP (50%–60% median LP/LNP) meta-analyses show a modest protective effect with summary odds ratios in both high LP and high LNP regions. This is much less evident and non-significant in mixed populations where lactase phenotype is not considered [118]. Figure 1 shows bar graph presentations of summary Odds Ratios (OR) or Relative Risks (RR) of regional meta-analyses of the effects of DFs on colorectal cancer.

In this analysis, confounding variables include the combined comparison of both case-control and cohort studies. In general, case-controlled studies offer less support for the protective effect of DFs on colorectal cancer and indeed most studies of mixed populations are based on this type. However, in the high LP or LNP populations, this discrepancy between methodologies has less of an influence. Other confounders include possible different lifestyles associated with DF consumption. However, if there were specific lifestyle associations with DF consumption, it is unclear why mixed populations should still show different results. Similarly, if different antitumor factors [111] in DFs were at play more in low LNP regions, it might still not explain results in mixed populations of LP/LNP.

Asian populations are predominantly LNP and consume less DFs than Western societies. Calcium intake on average from DFs may not be more than 1/3 of the intake compared with Western societies [8]. It is hypothesized that perhaps a prebiotic effect in LNP people compensates for this diminished calcium intake. Indirect support for this hypothesis is derived by the study of Roncucci *et al.*, who found a significant reduction in polyp formation after three years of lactulose ingestion [82]. Lactulose and lactose share several similar properties. Both are disaccharides. Lactulose is universally maldigested and malabsorbed while lactose has a somewhat similar role when existing intestinal lactase is overwhelmed. Both have been linked with induction of bifidobacteria in the colon and both have clinical benefits in treating hepatic encephalopathy. This condition is related to microbiome metabolism of ammonia.

A role for DFs in stomach cancer is fraught with inconsistencies with a recent meta-analysis finding no benefit of DFs in protection, and numerically that it may increase risk [119]. This review however follows three other reports (one is a meta-analysis from China) which supported a protective effect of dairy foods [120,121,122]. Another recent meta-analysis found total DFs reduce risk while milk has neutral effect [123]. The mechanisms have not been worked out yet and further investigations will be required.

A study evaluating diet in pancreatic cancer found that low fat DFs in conjunction with a diet low in meats, sugars and fats was protective in both men and women with similar relative risk [124]. Further studies are needed on pancreatic cancer and DF effects.

There is a suggestion that DFs may protect against bladder cancer [125,126,127] although this is controversial. It is also hypothesized that probiotics such as lactobacilli may have beneficial effects in superficial bladder cancer [128]. If this is true, a prebiotic effect of lactose from DFs may partly explain the modest benefits observed in the Japanese population [125]. Also such a postulate may explain a stronger inverse association with DFs noted in Asians compared with North Americans [126].

In several diseases, DF intake may increase risks for carcinogenesis. These diseases include ovarian [129], prostate [130,131] or testicular [132] cancers. Ovarian cancer has been consistently inversely related with lactase non persistence [133]. Initial hypotheses suggested that galactose could be toxic to ovaries [134]. However, this hypothesis was not confirmed, and meta-analyses of dietary studies report variable outcomes [135,136,137,138].

Dairy foods in prostate cancer seem to be consistently associated with increased risk. Calcium is hypothesized to play a role in carcinogenesis by interfering with Vitamin D metabolism and thereby increase risk [130,139]. As a result, increased consumption of DF would be expected to increase risk. However, the consumption of DFs in promoting prostate cancer has not been completely settled. For example, alleles for lactase persistence are not related to prostate cancer per se [131], however, others report decreased rates of cancer in some populations with increased LNP status [140]. In the most recent meta-analysis, factors other than calcium were hypothesized to be more relevant since supplemental calcium was not associated with increased risk [130].

A pathogenic role for calcium may also apply to testicular cancer [132]. In addition, the putative use of additives like estrogen in dairy foods or galactose itself has also been reported to be pathogenic in testicular cancer [141,142]. The observation that LP persons consume more DFs in general and the fact that less galactose is available for host utilization may reduce risks for ovarian and testicular cancers in LNP persons.

Several other cancers are found to occur more commonly in countries with higher per capita DF consumption. However, it is difficult to draw a connection on clinical grounds.

For breast cancer, initial studies suggested that risk may be increased by dairy foods [143], but early meta-analyses failed to substantiate an increased risk [144]. A recent meta-analysis suggested a modest protective effect of total dairy foods but not milk [145]. The included studies, however, proved to be heterogeneous, and as such there is still no clear consensus.

Lung cancer is most strongly associated with smoking. However, several studies suggested that DFs may contribute a deleterious effect to the cancer possibly through the effect of fat contained in DFs on lung [7,146]. There are no clear meta-analyses of this disease in relation to DFs.

A Swedish study found that a personal history of lactose intolerance was protective against lung, breast and ovarian cancer but this observation may reflect only personal diet [147]. The study is confounded the lack of distinction between LI/LNP and LI/LP.

There are also a number of other non-malignant diseases where the microbiome is thought to contribute to pathogenesis. Prominent among these are the inflammatory bowel diseases (IBD) [7]. However, the relationship specifically with DF consumption is conflicting. In Japan, DFs are reported to contribute to rising IBD rates [148]. In other reports, the contribution of DFs is also confusing [149,150]. Moreover, in an epidemiological study examining the role of mycobacterium avium paratuberculosis in Crohn’s disease, unpasteurized milk was found to be protective [151].

Reaction to lactose consumption varies between ulcerative colitis and Crohn’s disease where there is an increased LS independent of ethnicity [152]. This could be due to overt or subtle small bowel involvement. Furthermore, adaptation to the disaccharide lactulose was unsuccessful in patients with Crohn’s disease compared with healthy controls [153].This suggests a reason also why Crohn’s patients may have more difficulty with DFs containing lactose.

In obesity and the metabolic syndrome, the microbiome is thought to impact on pathogenesis (e.g., hypertension, type 2 diabetes [TD2], fatty liver). Although a meta-analysis of different prebiotics for obesity found reduced serum glucose and insulin with increased satiety no specific studies with lactose have been published [87]. DFs have been reported to show inverse associations with obesity and metabolic syndrome [154,155].

Relationship of DFs with diabetes is dichotomous. A detailed review of these interactions is beyond the scope of this article, however, there is a direct relationship with Type 1 DM (T1D) but an inverse relationship with Type 2 DM [156,157]. In this paradigm, T1D is thought to be an autoimmune disorder and not part of the metabolic syndrome as is Type 2 DM. Several components of DFs may be associated with the inverse association with T2D. These include the effect of calcium through Vitamin D metabolites, amino acids e.g., leucine, mitochondrial function, proteins such casein and whey or fatty acids. The review by Lacroix and Li-Chan concludes that the protective effects are likely to be non-specific to proteins since the inverse effect is also observed with fish and soy protein.

A meta-analysis of dairy foods in T2D is supportive of a protective effect before the onset of disease [158]. Table 7 summarizes these discussions dividing risks into diseases where DFs may decrease, show no significant effect or increase risks.

To date, however, the most important aspect of DF consumption involves its relationship with bone metabolism. However, this aspect will not be discussed other than to state that recent reviews emphasize this relationship [74]. Furthermore, the NIH conference on lactose intolerance in 2010 concluded that withdrawal of DFs from diet to treat LI leads to potential threats to bone health [11].

In the previous section on effects of pro and prebiotics, it is noted that there are many overlapping diseases. With the exception of one meta-analysis of colorectal cancer [118], a potential prebiotic effect of lactose in LNP persons has not been considered. The outcome of evaluating this prebiotic hypothesis is unknown. In this paradigm, it is not known whether colonic adaptation equalizes DF effects. This role may be exemplified by colon cancer where it was hypothesized that a prebiotic effect of lactose in LNP compensated for the calcium effect postulated in high DF intake/LP populations. Also, it is not known whether LNP persons who consume DFs regularly face different risks to LP persons with regards to several diseases (e.g., bladder cancer, obesity, T2D and possibly others). Ignoring the effects of lactose and the amount of lactose ingested may introduce confounders in studies examining the impact of DFs on different diseases.

**Table 7:** Summary of odds ratios or relative risks with 95% confidence. Intervals for different diseases related to consumption of Total Dairy Foods (TDF), milk or other milk products/nutrients are shown. Comparisons in studies were between highest and lowest intakes. Pancreatic, ovarian, and testicular cancer and obesity and Crohn’s disease are based on single studies. Colorectal, stomach, breast, bladder, prostate, and diabetes type II are based on meta-analyses. Population sample size is denoted by N. Origin refers to the source of the publication. Additional studies related to these diseases are discussed in the text.

Risk Decreased	No effect on Risk	Risk Increased
Colorectal Cancer [114] (19 cohort)	Ovarian [137] (cohort)	Prostate [130] (32 cohort)
TDF 0.83 (0.78–0.88)	Low fat DF 0.76 (0.54–1.06)	TDF 1.07 (1.02–1.12)
Milk 0.91 (0.85–0.94)	Lactose 0.87 (0.69–1.11) *	Milk 1.03 (1.00–1.07)
Cheese 0.96 (0.83–1.12)	N 764 women	N TDF; 38,107 of 848,395 participants Milk; 11,392 of 556,146 participants
NTDF; 11,579 cases *vs.* 1,170,942 controls Milk; 5011 cases *vs.* 655,483 controls	Origin United States	Origin United Kingdom
OriginUnited Kingdom		
Stomach [123] (17 case-control, 6 cohort)	Bladder [125] ** (14 studies)	Testicular [142] (case-control)
TDF 0.76 (0.64–0.91)	Milk 0.89 (0.77–1.02)	Milk 1.37 (1.12–1.68)
Not observed in Asians	N 7966 cases of 324,241 participants	Galactose 2.01 (1.41–2.86)
N 3256 cases *vs.* 17,026 controls	Origin China	N 269 cases *vs.* 797 controls
OriginChina		Origin Germany
Breast [145] (18 cohort)		
TDF 0.85 (0.76–0.95)		
N 24,187 cases *vs.* 163,471 controls		
Origin China		
Pancreas [124] (case-control)		
Low fat milk 0.51 (0.3–0.84)		
N 532 patients *vs.* 1701 controls		
Origin United States		
Crohn’s Disease [151] (case-control)		
Milk 0.82 (0.69–0.97)		
N 218 cases *vs.* 812 controls		
Origin United Kingdom		
Obesity [154] (nation-wide survey)		
TDF 0.51 (0.3–0.54)		
N 1352 cases		
Origin Luxembourg		
Dibetes Type II [158] (17 cohort)		
TDF		
N 22,877 cases *vs.* 526,487 controls		
Origin United Kingdom		

*: In this study [137] lactose intake varied inversely with Endometrioid epithelial ovarian carcinoma 0.32 (0.16–0.65) *p* < 0.001; **: In the case of Bladder Cancer the more negative meta-analysis is listed. A meta-analysis by Mao *et al.*, found a protective effect of milk intake which was statistically significantin Asia (OR 0.84(0.71–0.97) [126].

## 6. Summary

This paper reviews colonic adaptation to regular lactose consumption in LNP persons. Overall, despite controversy, the general medical literature has clouded the meaning of LI by involving causal conditions other than LM (e.g., systemic features, food sensitivities). In general, the treatment of LI may therefore vary depending on context.

However, evidence at this time suggests that in the LI/LM/LNP paradigm the regular consumption of DFs over a prolonged period (which may be as little a 3–4 weeks) improves aspects of LI symptoms. There are suggestions from the literature that over a prolonged time LI/LM/LNP persons may naturally become totally asymptomatic. The corollary is that an LNP person without LI who consumes regular DFs may be colonically adapted. How often this could happen in nature is not known. Symptomatic improvement may also be achieved by the use of other prebiotics, which could also allow more regular DF consumption.

LI/LM/LNP persons adapt through alterations of the microbiome/metabolome which are currently in the crosshairs of research. Numerous studies examine the possible effects of DFs on development of cancers and benign diseases. Yet, there are few studies which take into consideration the differential handling of lactose between LP/LNP populations evaluated for DF effects. The best examples are studies from Asia where populations might be more LNP homogenous. In studies of mixed populations, division into LP or LNP is much less clear. In some cases, ignoring such differences may confound outcomes possibly because mechanisms of DF effects could differ.

In this context, there are remaining questions about the lactase LP/LNP differences. These include: (1) universality of adaptation in LNP persons; (2) frequency of “deep (completely asymptomatic)” colonic adaptation; (3) confirmation of the quantity of regular DF consumption needed for metabolomic/metagenomic alterations in LNP persons; (4) possible differences in the metabolome/metagenome among LP, LP-DF consumers, LNP, and LNP-DF consumers; (5) whether long term DF intake in LNP modifies the effects on disease and whether these are similar or different from LP. Further investigations along these lines would be consistent with the emerging knowledge about the role of the microbiome and diseases pathogenesis.

Based on this review, DF consumption is encouraged based on age and type of DF. For example, adolescents are encouraged to consume DFs for development and bone health. Increased DFs also may intervene in the current epidemic of obesity and consequences of the metabolic syndrome. In specific diseases, as outlined, DFs may be beneficial (e.g., colon cancer). High DF intake in older men may be discouraged. However, for other diseases, more studies are needed to better define the risks.

## References

[1] Swallow D.M. (2003). Genetics of lactase persistence and lactose intolerance. Ann. Hum. Genet..

[2] Tishkoff S.A., Reed F.A., Ranciaro A., Voight B.F., Babbitt C.C., Silverman J.S. (2007). Convergent adaptation of human lactase persistence in Africa and Europe. Nat. Genet..

[3] Prentice A.M. (2014). Dairy products in global public health. Am. J. Clin. Nutr..

[4] Holden C., Mace R. (1997). Phylogenetic analysis of the evolution of lactose digestion in adults. Hum. Biol..

[5] Flatz G., Rotthauwe H.W. (1973). Lactose nutrition and natural selection. Lancet.

[6] Bersaglieri T., Sabeti P.C., Patterson N., Vanderploeg T., Schaffner S.F., Drake J.A. (2004). Genetic signatures of strong recent positive selection at the lactase gene. Am. J. Hum. Genet..

[7] Gerbault P., Liebert A., Swallow D.M., Thomas M.G., Lomer M. (2014). Lactose malabsorption and nutrition. Advanced Nutrition and Dietetics in Gastroenterology.

[8] Shrier I., Szilagyi A., Correa J.A. (2008). Impact of lactose containing foods and the genetics of lactase on diseases: An analytical review of population data. Cancer Nutr..

[9] Scrimshaw N.S., Murray E.B. (1988). Prevalence of lactose maldigestion. Am. J. Clin. Nutr..

[10] Hertzler S.R., Savaiano D.A. (1996). Colonic adaptation to daily lactose feeding in lactose maldigesters reduces lactose intolerance. Am. J. Clin. Nutr..

[11] Suchy F.J., Brannon P.M., Carpenter T.O., Fernandez J.R., Gilsanz V., Gould J.B., Hall K., Hui S.L., Lupton J., Mennella J. (2010). National Institutes of Health Consensus Development Conference: Lactose intolerance and health. Ann. Intern. Med..

[12] He T., Venema K., Priebe M.G., Welling G.W., Brummer R.J., Vonk R.J. (2008). The colonic metabolism in lactose intolerance. Eur. J. Clin. Investig..

[13] Shaukat A., Levitt M.D., Taylor B.C., MacDonald R., Shamliyan T.A., Kane R.L., Wilt T.J. (2010). Systematic review: Effective management strategies for lactose intolerance. Ann. Intern. Med..

[14] Eadala P., Waud J.P., Matthews S.B., Green J.T., Campbell A.K. (2009). Quantifying the “hidden” lactose in drugs used for the treatment of gastrointestinal conditions. Aliment. Pharmacol. Ther..

[15] Montalto M., Gallo A., Santoro L., D’Onofrio F., Curigliano V., Covino M., Cammarota G., Grieco A., Gasbarrini A., Gasbarrini G. (2008). Low-dose lactose in drugs neither increases breath hydrogen excretion nor causes gastrointestinal symptoms. Aliment. Pharmacol. Ther..

[16] Matthews S.B., Waud J.P., Roberts A.G., Campbell A.K. (2005). Systemic lactose intolerance: A new perspective on an old problem. Postgrad. Med. J..

[17] Eadala P., Matthews S.B., Waud J.P., Green J.T., Campbell A.K. (2011). Association of lactose sensitivity with inflammatory bowel disease—Demonstrated by analysis of genetic polymorphism, breath gases and symptoms. Aliment. Pharmacol. Ther..

[18] Gibson P.R., Shepherd S.J. (2010). Evidence-based dietary management of functional gastrointestinal symptoms: The FODMAP approach. J. Gastroenterol. Hepatol..

[19] Casellas F., Aparici A., Casaus M., Rodríguez P., Malagelada J.R. (2010). Subjective perception of lactose intolerance does not always indicate lactose malabsorption. Clin. Gastroenterol. Hepatol..

[20] Suarez F.L., Savaiano D.A., Levitt M.D. (1995). A comparison after the consumption of milk or lactose-hydrolyzed milk by people with self-reported severe lactose intolerance. N. Engl. J. Med..

[21] Suarez F.L., Savaiano D., Arbisi P., Levitt M.D. (1997). Tolerance to the daily ingestion of two cups of milk by individuals claiming lactose intolerance. Am. J. Clin. Nutr..

[22] Savaiano D.A., Boushey C.J., McCabe G.P. (2006). Lactose intolerance symptoms assessed by meta-analysis: A grain of truth that leads to exaggeration. J. Nutr..

[23] Vernia P., di Camillo M., Foglietta T., Avallone V.E., de Carolis A. (2010). Diagnosis of lactose intolerance and the “nocebo” effect: The role of negative expectations. Dig. Liver Dis..

[24] Briet F., Pochart P., Marteau P., Flourie B., Arrigoni E., Rambaud J.C. (1997). Improved clinical tolerance to chronic lactose ingestion in subjects with lactose intolerance: A placebo effect?. Gut.

[25] Habte D., Sterby G., Jijalmassen B. (1973). Lactose malabsorption in Ethiopian children. Acta Paediatr.Scand..

[26] Sadre M., Karbasi K. (1979). Lactose intolerance in Iran. Am. J. Clin. Nutr..

[27] Johnson A.O., Semenya J.G., Buchowski M.S., Enwonwu C.O., Scrimshaw N.S. (1993). Adaptation of lactose maldigesters to continued milk intakes. Am. J. Clin. Nutr..

[28] Villar J., Kestler E., Castillo P., Juarez A., Menendez R., Solomons N.W. (1988). Improved lactose digestion during pregnancy: A case of physiologic adaptation?. Obstet. Gynecol..

[29] Szilagyi A., Salomon R., Martin M., Fokeef K., Seidman E. (1996). Improved lactose handling in late phase pregnancy. Clin. Investig. Med..

[30] Paige D.M., Witter F.R., Bronner Y.L., Kessler L.A., Perman J.A., Paige T.R. (2003). Lactose digestion in pregnant African-Americans. Public Health Nutr..

[31] Ladas S., Papanikos J., Arapakis G. (1982). Lactose malabsorption in Greek adults: Correlation of small bowel transit time with the severity of lactose intolerance. Gut.

[32] Salomon R., Levy E., Levesque D., Szilagyi A., Seidman E. (1996). Caco-2 cell dissaccharidase activities are unaffected by gestational hormones. Can. J. Physiol. Pharmacol..

[33] Lazzara F., Szilagyi A., Lerman A. (1985). Lactose intolerance in hyperthyroidism: A possible unmasking effect. Am. J. Gastroenterol..

[34] Szilagyi A., Lerman S., Barr R.G., Stern J., MacMullan S. (1992). Influence of hypothyroidism on gastric emptying, oral cecal transit and lactose absorption. Clin. Investig. Med..

[35] Asik M., Gunes F., Binnetoglu E., Eroglu M., Bozkurt N., Sen H., Akbal E., Bakar C., Beyazit Y., Ukinc K. (2014). Decrease in TSH levels after lactose restriction in Hashimoto’s thyroiditis patients with lactose intolerance. Endocrine.

[36] Lebenthal A., Lebenthal E. (1999). The ontogeny of the small intestinal epithelium. J. Parenter. Enteral Nutr..

[37] Galluser M., Belkhou R., Freund J.N., Duluc I., Torp N., Danielsen M., Raul F. (1991). Adaptation of intestinal hydrolases to starvation in rats: Effect of thyroid function. J. Comp. Physiol. B.

[38] Szilagyi A., Lerman S., Barr R.G., Stern J., Colacone A., McMullan S. (1991). Reversible lactose malabsorption and intolerance in Graves’ disease. Clin. Investig. Med..

[39] Yaylali O., Kirac S., Yilmaz M., Akin F., Yuksel D., Demirkan N., Akdag B. (2009). Does hypothyroidism affect gastrointestinal motility?. Gastroenterol. Res. Pract..

[40] Daher R., Yazbeck T., Jaoude J.B., Abboud B. (2009). Consequences of dysthyroidism on the digestive tract and viscera. World J. Gastroenterol..

[41] Szilagyi A., Salomon R., Seidman E. (1996). Influence of Loperamide on lactose handling and oral caecal transit time. Aliment. Pharmacol. Ther..

[42] Szilagyi A., Rivard J., Fokeeff K. (2001). Improved parameters of lactose maldigestion using lactulose. Dig. Dis. Sci..

[43] Savaiano D.A., Ritter A.J., Klaenhammer T.B., James G.M., Longcore A.T., Chandler J.R., Walker W.A., Foyt H.L. (2013). Improving lactose digestion and symptoms of lactose intolerance with a novel galacto-oligosaccharide (RP-G28): A randomized, double-blind clinical trial. Nutr. J..

[44] Szilagyi A., Cohen A., Vinokuroff C., Ahmad D., Nathwani U., Yesovitch S. (2004). De-adapt and re-adapt with lactose but no cross-adapt to lactulose: A case of occult colonic bacterial adaptation. Can. J. Gastroenterol..

[45] Gibson G.R., Roberfroid M.B. (1995). Dietary modulation of the human colonic microbiota: Introducing the concept of prebiotics. J. Nutr..

[46] Morgan X.C., Huttenhower C. (2014). Meta’omic analytic techniques for studying the intestinal microbiome. Gastoeneterology.

[47] Hollister E.B., Gao X., Versalovic J. (2014). Compositional and functional features of gastrointestinal microbiome and their effects on human health. Gastroenterology.

[48] Carding S., Verbeke K., Vipond D.T., Corfe B.M., Owen L.J. (2015). Dysbiosis of the gut microbiota in disease. Microb. Ecol. Health Dis..

[49] Albenberg L.G., Wu G.D. (2014). Diet and the intestinal microbiome: Associations, functions, and implications for health and disease. Gastroenterology.

[50] Arumugam M., Raes J., Pelletier E.E., le Paslier D., Yamada T., Mende D.R., Fernandes G.R., Tap J., Bruls T., Batto J.M. (2011). Enterotypes of the human gut microbiome. Nature.

[51] Ritchie M.L., Romanuk T.N. (2012). A meta-analysis of probiotic efficacy for gastrointestinal diseases. PLoS ONE.

[52] Videlock E.J., Cremonini F. (2012). Meta-analysis: Probiotics in antibiotic-associated diarrhoea. Aliment. Pharmacol. Ther..

[53] Tung J.M., Dolovich L.R., Lee C.H. (2009). Prevention of Clostridium difficile infection with Saccharomyces boulardii: A systematic review. Can. J. Gastroenterol..

[54] Hoveyda N., Heneghan C., Mahtani K.R., Perera R., Roberts N., Glasziou P. (2009). A systematic review and meta-analysis: Probiotics in the treatment of irritable bowel syndrome. BMC Gastroenterol..

[55] Shen J., Zuo Z.X., Mao A.P. (2014). Effect of probiotics on inducing remission and maintaining therapy in ulcerative colitis, Crohn’s disease, and pouchitis: Meta-analysis of randomized controlled trials. Inflamm. Bowel Dis..

[56] Michail S.K., Stolfi A., Johnson T., Onady G.M. (2008). Efficacy of probiotics in the treatment of pediatric atopic dermatitis: A meta-analysis of randomized controlled trials. Ann. Allergy AsthmaImmunol..

[57] Lindsay K.L., Walsh C.A., Brennan L., McAuliffe F.M. (2013). Probiotics in pregnancy and maternal outcomes: A systematic review. J. Matern. Fetal Neonatal Med..

[58] Doege K., Grajecki D., Zyriax B.C., Detinkina E., Zu Eulenburg C., Buhling K.J. (2012). Impact of maternal supplementation with probiotics during pregnancy on atopic eczema in childhood: A meta-analysis. Br. J. Nutr..

[59] Razmpoosh E., Javadi M., Ejtahed H.S., Mirmiran P. (2015). Probiotics as beneficial agents on the management of diabetes mellitus: A systematic review. Diabetes Metab. Res. Rev..

[60] Almeida C.C., Lorena S.L., Pavan C.R., Akasaka H.M., Mesquita M.A. (2012). Beneficial effects oflong-term consumption of a probiotic combination of Lactobacillus casei Shirota and Bifidobacteriumbreve Yakult may persist after suspension of therapy in lactose-intolerant patients. Nutr. Clin. Pract..

[61] Malolepszy P., Shrier I., Szilagyi A. (2006). Adaptation to lactose intolerance is not achieved by long term ingestion of a multi species containing probiotic. Int. J. ProbioticsPrebiotics..

[62] Macfarlane S., Macfarlane G.T., Cummings J.H. (2006). Review Article: Prebiotics in the gastrointestinal tract. Aliment. Pharmacol. Ther..

[63] Ursell L.K., Haiser H.J., van Treuren W., Garg N., Reddivan L., Vanamala J., Dorrestein P.C., Turnbaugh P.J., Knight R. (2014). The intestinal metabolome: An intersection between microbiota and host. Gastroenterology.

[64] McNulty N.P., Yatsunenko T., Hsiao A., Faith J.J., Muegge B.D., Goodman A.L., Henrissat B., Oozeer R., Cools-Portier S., Gobert G. (2011). The impact of a consortium of fermented milk strains on the gut microbiome of gnotobiotic mice and monozygotic twins. Sci. Transl. Med..

[65] Young S.L., Simon M.A., Baird M.A., Tannock G.W., Bibiloni R., Spencely K., Lane J.M., Fitzharris P., Crane J., Town I. (2004). Bifidobacterial species differentially affect expression of cell surface markers and cytokines of dendritic cells harvested from cord blood. Clin. Diagn. Lab. Immunol..

[66] De Filippo C., Cavalieri D., di Paola M., Ramazzotti M., Poullet J.B., Massart S., Collini S., Pieraccini G., Lionetti P. (2010). Impact of diet in shaping gut microbiota revealed by a comparative study in children from Europe and rural Africa. Proc. Natl. Acad. Sci. USA.

[67] Slavin J. (2013). Fiber and prebiotics: Mechanisms and health benefits. Nutrients.

[68] Hamer H.M., Jonkers D., Venema K., Vanhoutvin S., Troost J., Brummer R.-J. (2008). Review article: The role of butyrate on colonic function. Aliment. Pharmacol. Ther..

[69] Frei R., Akdis M., O’Mahony P. (2015). Prebiotics, probiotics, synbiotics and the immune system: Experimental data and clinical evidence. Curr. Opin. Gastroenterol..

[70] Bouhnik Y., Attar A., Joly F.A., Riottot M., Dyard F., Flourié B. (2004). Lactulose ingestion increases faecal bifidobacterial counts: A randomised double-blind study in healthy humans. Eur. J. Clin. Nutr..

[71] Falony G., Lazidou K., Verschaeren A., Weckx S., Maes D., de Vuyst L. (2009). *In vitro* analysis of fermentation of prebiotic inulin-type fructans by bifidobacterium species reveals four different phenotypes. Appl. Environ. Microbiol..

[72] Falony G., Calmeyn T., Leroy F., de Vuyst L. (2009). Coculture fermentations of bifidobacterium species and bacteroides thetaiotaomicron reveal a mechanistic insight into the prebiotic effect of inulin-type fructans. Appl. Environ. Microbiol..

[73] Duncan S.H., Louis P., Flint H.J. (2004). Lactate—Utilizing bacteria, isolated from human feces, that produce butyrate as a major fermentation product. Appl. Environ. Microbiol..

[74] Rizzoli R. (2014). Dairy products, yogurts, and bone health. Am. J. Clin. Nutr..

[75] Weichert S., Schroten H., Adam R. (2012). The role of prebiotics in prevention and treatment of childhood infectious diseases. Pediatr. Infect. Dis. J..

[76] Ford A.C., Quigley E.M.M., Lacy B.E., Lembo A.J., Saito Y.A., Schiller L.R., Soffer E.E., Spiegel B.M.R., Moayyedi P. (2014). Efficacy of prebiotics, probiotics and synbiotics in irritable bowel syndrome and chronic idiopathic constipation: Systematic review and meta-analysis. Am. J. Gastroenterol..

[77] Paineau D., Payen F., Panserieu S., Coulombier G., Sobaszek A., Lartigau I., Brabet M., Galmiche J.P., Tripodi D., Sacher-Huvelin S. (2008). The effects of regular consumption of short-chainfructo-oligosaccharides on digestive comfort of subjects with minor functional bowel disorders. Br. J. Nutr..

[78] Silk D.B., Davis A., Vulevic J., Tzortzis G., Gibson G.R. (2009). Clinical trial: The effects of a trans-galacto-oligosaccharide prebiotic on faecal microbiota and symptoms in irritable bowel syndrome. Aliment. Pharmacol. Ther..

[79] Ghouri Y.A., Richards D.M., Rahimi E.F., Krill J.T., Jelinek K.A., DuPont A.W. (2014). Systematic review of randomized controlled trials of probiotics, prebiotics and synbiotics in inflammatory bowel disease. Clin. Exp. Gastroenterol..

[80] Raman M., Ambalam P., Kondepudi K.K., Pithva S., Kothan C., Patel A.T., Purama R.K., Dave J.M., Vyas B.R.M. (2013). Potential of probiotics, prebiotics and synbiotics for management of colorectal cancer. Gut Microbes.

[81] Clark M.J., Robien K., Slavin J.L. (2012). Effector prebiotics on biomarkers of colorectal cancer in humans: A systematic review. Nutr. Rev..

[82] Roncucci L., di Donato P., Carati L., Ferrari A., Perini M., Bertoni G., Bedogni G., Paris B., Svanoni F., Girola M. (1993). Antioxidant vitamins or lactulose for the prevention of the recurrence of colorectal adenomas. Dis. Colon Rectum.

[83] Patel R.M., Denning P.W. (2013). Therapeutic use of prebiotics, probiotics and postbiotics to prevent necrotizing enterocolitis: What is the current evidence?. Clin. Perinatol..

[84] Uribe M., Márquez M.A., Garcí-Ramos G., Escobedo V., Murillo H., Guevara L., Lisker R. (1980). Treatment of chronic portal-systemic encephalopathy with lactose in lactase-deficient patients. Dig. Dis. Sci..

[85] Uribe M., Toledo H., Perez F., Vargas F., Gil S., Garcia-Ramos G., Ravelli G.P., Guevara L. (1987). Lactitol, a second-generation disaccharide for treatment of chronic portal-systemic encephalopathy. A double-blind, crossover, randomized clinical trial. Dig. Dis. Sci..

[86] Wen J., Liu Q., Song J., Tong M., Peng T., Liang H. (2013). Lactulose is highly potential in prophylaxis of hepatic encephalopathy in patients with cirrhosis and upper gastrointestinal bleeding: Results of a controlled randomized trial. Digestion.

[87] Kellow N.J., Coughlan M.T., Reid C.M. (2014). Metabolic benefits of dietary prebiotics in human subjects: A systematic review of randomized controlled trials. Br. J. Nutr..

[88] Osborn D.A., Sinn J.K. (2013). Prebiotics in infants for prevention of allergy. Cochrane Database Syst. Rev..

[89] Baquerizo Nole K.L., Yim E., Keri J.E. (2014). Probiotics and prebiotics in dermatology. J. Am. Acad. Dermatol..

[90] Battle M., Martin T., Fulton J. (2001). Lactulose may help prevent urinary tract infections. Br. Med. J..

[91] Hamer H.M., Jonkers D.M., Vanhoutvin S.A., Troost F.J., Rijkers G., de Bruïne A., Bast A., Venema K., Brummer R.J. (2010). Effect of butyrate enemas on inflammation and antioxidant status in the colonic mucosa of patients with ulcerative colitis in remission. Clin. Nutr..

[92] Guillemot F., Colombel J.F., Neut C., Verplanck N., Lecomte M., Romond C., Paris J.C., Cortot A. (1991). Treatment of diversion colitis by short-chain fatty acids. Prospective and double-blind study. Dis. Colon Rectum.

[93] Jiang T., Savaiano D.A. (1997). *In vitro* lactose fermentation by human colonic bacteria is modified by lactobacillus acidophilus supplementation. J. Nutr..

[94] Jiang T., Savaiano D.A. (1997). Modification of colonic fermentation by bifidobacteria and pH in vitro (Impact on lactose metabolism, short-chain fatty acid, and lactate production). Dig. Dis. Sci..

[95] Uribe-Esquivel M., Maran S., Poo J.L., Munoz R.M. (1997). *In vitro* and *in vivo* lactose and lactulose effects on colonic fermentation and portal-systemic encephalopathy parameters. Scand. J. Gastroenterol..

[96] Szilagyi A. (2004). Redefining lactose as a conditional prebiotic. Can. J. Gastroenterol..

[97] Gilat T., Russo S., Gelman-Malachi E., Aldor T.A. (1972). Lactase in man: A nonadaptable enzyme. Gastroenterology.

[98] Keusch G.T., Troncale F.J., Thavaramara B., Prinyanont P., Anderson P.R., Bhamarapravathi N. (1969). Lactase deficiency in Thailand : Effects of prolonged lactose feeding. Am. J. Clin. Nutr..

[99] Bond J.H., Levitt M.D. (1976). Quantitative measurement of lactose absorption. Gastroenterology.

[100] Hertzler S.R., Savaiano D.A., Levitt M.D. (1997). Fecal hydrogen production and consumption measurements. Response to daily lactose ingestion by lactose maldigesters. Dig. Dis. Sci..

[101] Makivuokko H.A., Saarinen M.T., Ouwehand A.C., Rautonen N.E. (2006). Effects of lactose on colon microbial community structure and function in a four-stage semi-continuous culture system. Biosci. Biotechnol. Biochem..

[102] Sanz M.L., Gibson G.R., Rastall R.A. (2005). Influence of disaccharide structure on prebiotic selectivity *in vitro*. J. Agric. Food Chem..

[103] Daly K., Darby A.C., Hall N., Nau A., Bravo D., Shirazi-Beechey S.P. (2014). Dietary supplementation with lactose or artificial sweetener enhances swine gut lactobacillus population abundance. Br. J. Nutr..

[104] Alexandre V., Even P.C., Larue-Achagiotis C., Blouin J.M., Blachier F., Benamouzig R., Tome D., Davila A.-M. (2013). Lactose absorption and colonic fermentation after host metabolism in rats. Br. J. Nutr..

[105] Ito M., Kimura M. (1993). Influence of lactose on faecal microflora in lactose maldigesters. Microb. Ecol. Health Dis..

[106] Szilagyi A., Shrier I., Heilpern D., Je J.S., Park S.H., Chong G., Lalonde C., Cote L.-F., Lee B.H. (2010). Differential impact of lactose/lactase phenotype on colonic microflora. Can. J. Gastroenterol..

[107] Palframan R., Gibson G.R., Rastall R.A. (2003). Development of a quantitative tool for the comparison of the prebiotic effect of dietary oligosaccharides. Lett. Appl. Microbiol..

[108] Oku T., Nakamura S., Ichinose M. (2005). Maximum permissive dose of lactose and lactitol for transitory diarrhea, and utilizable capacity for lactose in Japanese female adults. J. Nutr. Sci. Viaminol..

[109] Hertzler S.R., Huynh B.C., Savaiano D.A. (1996). How much lactose is low lactose?. J. Am. Diet. Assoc..

[110] Bouhnik Y., Vahedi K., Achour L., Attar A., Salfati J., Pochart P., Marteau P., Flourié B., Bornet F., Rambaud J.C. (1999). Short-chain fructo-oligosaccharide administration dose-dependently increases fecal bifidobacteria in healthy humans. J. Nutr..

[111] Pereira P.C. (2014). Milk nutritional composition and its role in human health. Nutrition.

[112] Park Y., Leitzmann M.F., Subar A.F., Hollenbeck A., Schatzkin A. (2009). Dairy Food, Calcium, and Risk of Cancer in the NIH-AARP Diet and Health Study. Arch. Intern. Med..

[113] Cho E., Smith-Warner S.A., Spiegelman D., Beeson W.L., van den Brandt P.A., Colditz G.A., Folsom A.R., Fraser G.E., Freudenheim J.L., Giovannucci E. (2004). Dairy foods, calcium, and colorectal cancer: A pooled analysis of 10 cohort studies. J. Natl. Cancer Inst..

[114] Aune D., Lau R., Chan D.S., Vieira R., Greenwood D.C., Kampman E., Norat T. (2012). Dairy products and colorectal cancer risk: A systematic review and meta-analysis of cohort studies. Ann. Oncol..

[115] Weingarten M.A., Zalmanovici Trestioreanu A., Yaphe J. (2008). Dietary calcium supplementation for preventing colorectal cancer and adenomatous polyps. Cochrane Database Syst. Rev..

[116] Chandler P.D., Buring J.E., Manson J.E., Giovannucci E.L., Moorthy M.V., Zhang S., Lee I.M., Lin J.H. (2015). Circulating Vitamin D Levels and Risk of Colorectal Cancer in Women. Cancer Prev. Res..

[117] Bostick R.M. (2015). Effects of supplemental vitamin D and calcium on normal colon tissue and circulating biomarkers of risk for colorectal neoplasms. J. Steroid Biochem. Mol. Biol..

[118] Szilagyi A., Nathwani U., Vinukoroff C., Correa A., Shrier I. (2006). The effect of lactose maldigestion on the relationship between dairy food intake and colorectal cancer: A systematic review. Nutr. Cancer.

[119] Tian S.B., Yu J.C., Kang W.M., Ma Z.Q., Ye X., Cao Z.J. (2014). Association between dairy intake and gastric cancer: A meta-analysis of observational studies. PLoS ONE.

[120] Lee H.H., Wu H.Y., Chuang Y.C., Chang A.S., Chao H.H., Chen K.Y., Chen H.K., Lai G.M., Huang H.H., Chen C.J. (1990). Epidemiologic characteristics and multiple risk factors of stomach cancer in Taiwan. Anticancer Res..

[121] Pham T.M., Fujino Y., Kikuchi S., Tamakoshi A., Matsuda S., Yoshimura T. (2010). Dietary patterns and risk of stomach cancer mortality: The Japan collaborative cohort study. Ann. Epidemiol..

[122] Huang Y.X., Qin L.Q., Wang P.Y. (2009). Meta-analysis of the relationship between dairy product consumption and gastric cancer. Zhonghua Yu Fang Yi Xue Za Zhi.

[123] Guo Y., Shan Z., Ren H., Chen W. (2015). Dairy consumption and gastric cancer risk: A meta-analysisof epidemiological studies. Nutr. Cancer.

[124] Chan J.M., Gong Z., Holly E.A., Bracci P.M. (2013). Dietary patterns and risk of pancreatic cancer in a large population-based case-control study in the San Francisco Bay Area. Nutr. Cancer.

[125] Li F., An S.L., Zhou Y., Liang Z.K., Jiao Z.J., Jing Y.M., Wan P., Shi X.J., Tan W.L. (2011). Milk and dairy consumption and risk of bladder cancer: A meta-analysis. Urology.

[126] Mao Q.Q., Dai Y., Lin Y.W., Qin J., Xie L.P., Zheng X.Y. (2011). Milk consumption and bladder cancer risk: A meta-analysis of published epidemiological studies. Nutr. Cancer.

[127] Keszei A.P., Schouten L.J., Goldbohm R.A., van den Brandt P.A. (2010). Dairy intake and the risk of bladder cancer in the Netherlands Cohort Study on Diet and Cancer. Am. J. Epidemiol..

[128] Feyisetan O., Tracey C., Hellawell G.O. (2012). Probiotics, dendritic cells and bladder cancer. BJU Int..

[129] Larsson S.C., Orsini N., Wolk A. (2006). Milk, milk products and lactose intake and ovarian cancer risk: A meta-analysis of epidemiological studies. Int. J. Cancer.

[130] Aune D., Navarro Rosenblatt D.A., Chan D.S., Vieira A.R., Vieira R., Greenwood D.C., Vatten L.J., Norat T. (2015). Dairy products, calcium, and prostate cancer risk: A systematic review and meta-analysis of cohort studies. Am. J. Clin. Nutr..

[131] Travis R.C., Appleby P.N., Siddiq A., Allen N.E., Kaaks R., Canzian F., Feller S., Tjønneland A., Føns Johnsen N., Overvad K. (2013). Genetic variation in the lactase gene, dairy product intake and risk for prostate cancer in the European prospective investigation into cancer and nutrition. Int. J. Cancer.

[132] Garner M.J., Birkett N.J., Johnson K.C., Shatenstein B., Ghadirian P., Krewski D. (2003). Canadian Cancer Registries Epidemiology Research Group. Dietary risk factors for testicular carcinoma. Int. J. Cancer.

[133] Cramer D.W. (1989). Lactase persistence and milk consumption as determinants of ovarian cancer risk. Am. J. Epidemiol..

[134] Cramer D.W., Harlow B.L., Willett W.C., Welch W.R., Bell D.A., Scully R.E., Ng W.G., Knapp R.C. (1989). Galactose consumption and metabolism in relation to the risk of ovarian cancer. Lancet.

[135] Faber M.T., Jensen A., Søgaard M., Høgdall E., Høgdall C., Blaakaer J., Kjaer S.K. (2012). Use of dairy products, lactose, and calcium and risk of ovarian cancer—Results from a Danish case-controlstudy. Acta Oncol..

[136] Genkinger J.M., Hunter D.J., Spiegelman D., Anderson K.E., Arslan A., Beeson W.L., Buring J.E., Fraser G.E., Freudenheim J.L., Goldbohm R.A. (2006). Dairy products and ovarian cancer: A pooled analysis of 12 cohort studies. Cancer Epidemiol. Biomarker. Prev..

[137] Merritt M.A., Poole E.M., Hankinson S.E., Willett W.C., Tworoger S.S. (2014). Dairy food and nutrient intake in different life periods in relation to risk of ovarian cancer. Cancer Causes Control.

[138] Merritt M.A., Cramer D.W., Vitonis A.F., Titus L.J., Terry K.L. (2013). Dairy foods and nutrients in relation to risk of ovarian cancer and major histological subtypes. Int. J. Cancer.

[139] Skowronski R.J., Peehl P.Y., Feldman D. (1993). Vitamin D and prostate cancer: 1,25 dihydroxyvitamin D3 receptors and actions in human prostate cancer cell lines. Endocrinology.

[140] Agarwal M.M., Rana S.V., Mandal A.K., Malhotra S., Khandelwal N., Kumar S., Acharya N.C., Singh S.K. (2008). Lactose intolerance in prostate cancer patients: Incidence and associated factors. Scand. J. Gastroeneterol..

[141] Davies T.W., Palmer C.R., Lipscombe J.M. (1998). Adolescent milk, dairy product and fruit consumption and testicular cancer. Br. J. Cancer.

[142] Stang A., Ahrens W., Baumgrdt-Elms C. (2006). Adolescent milk fat and galactose consumption and testicular cell cancer. Cancer Epidemiol. Biomark. Prev..

[143] Ganmaa D., Sato A. (2005). The possible role of female sex hormones in milk from pregnant cows in the development of breast, ovarian and corpus uteri cancers. Med. Hypotheses.

[144] Pala V., Krogh V., Berrino F., Sieri S., Grioni S., Tjønneland A., Olsen A., Jakobsen M.U., Overvad K., Clavel-Chapelon F. (2009). Meat, eggs, dairy products, and risk of breast cancer in the European Prospective Investigation into Cancer and Nutrition (EPIC) cohort. Am. J. Clin. Nutr..

[145] Dong J.Y., Zhang L., He K., Qin L.Q. (2011). Dairy consumption and risk of breast cancer: A meta-analysis of prospective cohort studies. Breast Cancer Res. Treat..

[146] Sankaranarayanan R., Varghese C., Duffy S.W., Padmakumary G., Day N.E., Nair M.K. (1994). A case-control study of diet and lung cancer in Kerala, south India. Int. J. Cancer.

[147] Ji J., Sundquist J., Sundquist K. (2015). Lactose intolerance and risk of lung, breast and ovarian cancers: Aetiological clues from a population-based study in Sweden. Br. J. Cancer.

[148] Asakura H., Suzuki K., Kitahora T., Morizane T. (2008). Is there a link between food and intestinal microbes and the occurrence of Crohn’s disease an ulcerative colitis. J. Gastroenterol. Hepatol..

[149] Joachim G. (1999). The relationship between habits of food consumption and reported reactions to food in people with inflammatory bowel disease—Testing the limits. Nutr. Health.

[150] Jantchou P., Morois S., Clavel-Chapelon F., Boutron-Ruault M.C., Carbonnel F. (2010). Animal protein intake and risk of inflammatory bowel disease: The E3N prospective study. Am. J. Gastroenterol..

[151] Abubakar I., Myhill D.J., Hart A.R., Lake I.R., Harvey I., Rhodes J.M., Robinson R., Lobo A.J., Probert C.S., Hunter P.R. (2007). A case-control study of drinking water and dairy products in Crohn’s disease—Further investigation of the possible role of Mycobacterium avium paratuberculosis. Am. J. Epidemiol..

[152] Mishkin B., Yalovsky M., Mishkin S. (1997). Increased prevalence of lactose malabsorption in Crohn’s disease patients at low risk for lactose malabsorption based on ethnic origin. Am. J. Gastroenterol..

[153] Szilagyi A., Rivard J., Shrier J. (2002). Diminished efficacy of colonic adaptation to lactulose occurs in patients with inflammatory bowel disease in remission. Dig. Dis. Sci..

[154] Crichton G.E., Alkerwi A. (2014). Whole-fat dairy food intake is inversely associated with obesity prevalence: Findings from the Observation of Cardiovascular Risk Factors in Luxembourg study. Nutr. Res..

[155] O’Sullivan T.A., Bremner A.P., Bremer H.K., Seares M.E., Beilin L.J., Mori T.A., Lyons-Wall P., Devine A., Oddy W.H. (2014). Dairy product consumption, dietary nutrient and energy density and associations with obesity in Australian adolescents. J. Hum. Nutr. Diet..

[156] Hirahatake K.M., Slavin J.L., Maki K.C., Adams S.H. (2014). Associations between dairy foods, diabetes, and metabolic health: Potential mechanisms and future directions. Metabolism.

[157] Lacroix I.M., Li-Chan E.C. (2014). Investigation of the putative associations between dairy consumption and incidence of type 1 and type 2 diabetes. Crit. Rev. Food Sci. Nutr..

[158] Aune D., Norat T., Romundstad P., Vatten L.J. (2013). Dairy products and the risk of type 2 diabetes: A systematic review and dose-response meta-analysis of cohort studies. Am. J. Clin. Nutr..

